# Identification of novel genes in Behcet’s disease using integrated bioinformatic analysis

**DOI:** 10.1007/s12026-022-09270-3

**Published:** 2022-04-02

**Authors:** Si Chen, Haolong Li, Haoting Zhan, Xiaoli Zeng, Hui Yuan, Yongzhe Li

**Affiliations:** 1 Department of Clinical Laboratory, State Key Laboratory of Complex Severe and Rare Diseases, Dongcheng District, Peking Union Medical College Hospital, Chinese Academy of Medical Science and Peking Union Medical College, 1 Shuaifuyuan, Beijing, 100730 People’s Republic of China; 2 Department of Clinical Laboratory, Beijing Anzhen Hospital, Capital Medical University, Beijing, People’s Republic of China

**Keywords:** Behcet’s disease, Microarray, Differentially expressed genes, Robust rank aggregation, Integrated analysis

## Abstract

Behcet’s disease (BD) is a chronic vascular inflammatory disease. However, the etiology and molecular mechanisms underlying BD development have not been thoroughly understood. Gene expression data for BD were obtained from the Gene Expression Omnibus database. We used robust rank aggregation (RRA) to identify differentially expressed genes (DEGs) between patients with BD and healthy controls. Gene ontology functional enrichment was used to investigate the potential functions of the DEGs. Protein–protein interaction (PPI) network analysis was performed to identify the hub genes. Receiver operating characteristic analyses were performed to investigate the value of hub genes in the diagnosis of BD. GSE17114 and GSE61399 datasets were included, comprising 32 patients with BD and 26 controls. The RRA integrated analysis identified 44 significant DEGs among the GSE17114 and GSE61399 CD4 + T lymphocytes. Functional enrichment analysis revealed that protein tyrosine/threonine phosphatase activity and immunoglobulin binding were enriched in BD. PPI analysis identified *FCGR3B* as a hub gene in the CD4 + T lymphocytes of BD patients. Our bioinformatic analysis identified new genetic features, which will enable further understanding of the pathogenesis of BD.

**Supplementary Information:**

The online version contains supplementary material available at 10.1007/s12026-022-09270-3.

## Introduction

Behcet’s disease (BD) is a chronic recurrent vascular inflammatory disease that can involve all types of blood vessels throughout the mouth, skin, genitals, eyes, and important organs of the cardiovascular system, digestive tract, nervous system, and joints [1]. The distribution of BD exhibits distinct ethnic and regional differences. The prevalence of BD is high in the Mediterranean coast, the Middle East, and Southeast Asia, namely the “Silk Road” region, and low in Europe and America. The prevalence of BD in China is 14.0/100,000, which is very similar to that in Japan (13.5/100,000) [2]. However, the pathogenesis of BD is not clear, and previous studies have shown that the incidence of BD is mainly related to autoimmune, environmental, and genetic factors [3]. Fei et al. conducted the first genome-wide association study (GWAS) of BD in Turkish population. Although this study did not identify any significant loci at the GWAS level, it was a landmark study in understanding the genetics of BD [4]. To date, a total of 21 genetic susceptibility sites for BD have been identified at the GWAS significance level, including interleukin-23 receptor (*IL23R*) and interleukin-10 (*IL10*) [5]. Related studies have shown that several immune cells, such as natural killer cells, monocytes, and B cells play an important role in the pathogenesis of BD [6]. The number of CD4^+^ and CD8^+^ T cells increased in circulating blood and inflammatory tissues of BD [7–9]; Th1 and Th17 cell numbers increased and caused inflammation in the early stage of BD intestinal involvement [10]. The study of Immunochip array [11] and genotyping array [12–16] in BD showed that immune-mediated and genetic factors were key in its pathogenesis. Some novel susceptible genes, such as interferon γ receptor 1 (*IFNGR1*) [17], have been identified. Meanwhile, gene microarray technology [18, 19] has been used to analyze the expression of genes in the peripheral blood mononuclear cells of BD patients. However, the results of these microarrays are not ideal, owing to differences in analysis methods and sample sources. Bioinformatic analysis is an effective method for in-depth detection and mining of transcriptome data and is widely used in various diseases [20–22]. In this study, two mRNA microarray datasets were screened using the GEO database. In robust rank aggregation (RRA) analysis, the data were grouped according to CD14 + monocytes and CD4 + T lymphocytes to identify differentially expressed genes (DEGs). Subsequently, we used gene ontology (GO) function enrichment analysis to explore the molecular mechanisms underlying BD. Protein–protein interaction (PPI) network analysis was used to screen for key genes. Finally, a validation test was conducted to determine the key hub genes involved in the pathogenesis of BD. This study aimed to discover new DEGs involved in BD pathogenesis and explore the possible molecular mechanisms associated with CD4 + T lymphocytes in BD.

## Materials and methods

### Study design and data collection

GEO (http://www.ncbi.nlm.nih.gov/geo) is a common database that hosts microarray, high-throughput sequencing, and chip data [23], and we employed it to search the related gene expression data using the following terms: “Behçet’s disease,” “Vasculitis,” “Gene expression,” “*Homo sapiens*,” and “Microarray.” The following inclusion criteria were used: (1) involvement of more than ten specimens; (2) total RNA was extracted from peripheral blood mononuclear cells; (3) gene expression data in CEL format were obtained from GEO; and finally, GSE17114 and GSE61399 [18] were selected. We used the “affy” package [24] for background correction, the “gcrma” package [25] for standardized processing, the “sva” package [26] to remove batch effect, and the “rsubread” package [27] for gene annotation. For comparing data before and after standardization, we used a box chart for visualization. Meanwhile, comparing data before and after removing the batch effect, we used principal component analysis (PCA) for visualization. In gene annotation, we had the following rules: (1) the average value of multiple probes matching the same genetic symbol was used and (2) genes or probes without corresponding genetic signs were deleted.

### Differentially expressed gene screening

We divided the two GEO datasets into three different groups, because the GSE61399 dataset comprised CD14 + monocytes and CD4 + T lymphocytes. We performed differential analysis using the “limma” package [28] to detect DEGs between BD and healthy controls, set *P* values ≤ 0.05 and |log_2_ fold change (FC)|≥ 0.5 as significant, and used the “ggplot2” package [29] to map the volcano.

### RRA analysis

RRA is an effective tool for combining the results [30]. To reduce the differences and combine multiple microarray results, RRA analysis was used to identify typical DEGs. The specific steps of analysis were as follows: First, by analyzing the expression of FC between BD and control, we obtained the lists of upregulated and downregulated genes in each dataset. Second, we used the “robust rank aggregation” package [30] to aggregate the list of all sequenced genes in the datasets. We used the Benjamin and Hochberg false discovery rate (FDR) method to generate the adjusted *P*-value and screened the significant genes with adjusted *P* < 0.5 and |log_2_FC|> 0.5.

### Functional enrichment analysis

In order to investigate the role of DEGs in the pathogenesis of BD, we used the “clusterprofiler” package [31] to conduct GO functional enrichment analysis of important genes identified by RRA. In addition, we used the “clusterprofiler” package (cnetplot) for visualization. Our criteria were adjusted at *P* < 0.05 and the false discovery rate (FDR) < 0.05.

### PPI network analysis

STRING is an online database for predicting PPI [32]. First, we fed important genes from the above RRA analysis into the STRING database. Second, the results of STRING analysis with an intermediate confidence of > 0.4 were collected. Third, we exported the TSV format data to the Cytoscape software (version 3.7.2) that is used to visualize the PPI network [33].

### Diagnostic effectiveness evaluation

For diagnostic analysis, we selected GSE17114, GSE61399, and GSE61399 CD4 + T lymphocytes. We chose the data of this study for verification because GSE165254 [34] is sequencing data and the original data of GSE165254 cannot be obtained. GSE70403 [19] only included patients with BD, not healthy controls. The receiver operator characteristic (ROC) curves were diagramed and the area under curve (AUC) was measured to appraise the performance of each dataset (GSE17114, GSE61399, and GSE61399 CD4 + T lymphocytes) using the “pROC” package in R [35]. We defined the criteria to distinguish between different diagnostic values as follows: excellent accuracy (0.9 ≤ *AUC* < 1), reasonable accuracy (0.8 ≤ *AUC* < 0.9), fair accuracy (0.7 ≤ *AUC* < 0.8), poor accuracy (0.6 ≤ *AUC* < 0.7), and insufficient accuracy (0.5 ≤ *AUC* < 0.6) [36].

## Results

### Information of included microarrays

According to the previously established inclusion criteria, GSE17114 and GSE61399 were included in this study; 32 BD patients and 26 controls were included in these two datasets. The clinical data of GSE17114 were relatively integrated, including 15 BD patients (women, 53.3%; mean age, 37.07 ± 10.67 years; immunosuppressors, 60.0%) and 14 healthy controls (women, 50.0%; mean age, 36.71 ± 13.00 years); however, the clinical data of GSE61399 did not provide information for the 17 BD patients and 12 healthy controls. The analyses of GSE17114 and GSE61399 series were performed on the GPL570 platform (Affymetrix Human Genome U133 Plus 2.0 Array). The RNA of the GSE17114 dataset was derived from peripheral blood mononuclear cells, whereas the RNA from the GSE61399 dataset was derived from CD14 + monocytes and CD4 + T lymphocytes. Detailed information on these datasets is shown in Table 1.

**Table 1:** Characteristics of the enrolled microarray datasets

GSE ID	BD	Control	Tissues	Analysis type	Platform	Citation (PMID)	Country	Year
GSE17114	15	14	Peripheral blood mononuclear cells	Array	GPL570 [HG-U133_Plus_2] Affymetrix Human	No	Portugal	2019
GSE61399	17	12	CD14 + monocytes and CD4 + T lymphocytes	Array	GPL570 [HG-U133_Plus_2] Affymetrix Human	25,410,656	USA	2014

### Identification of DEGs in BD

**Fig. 1:**
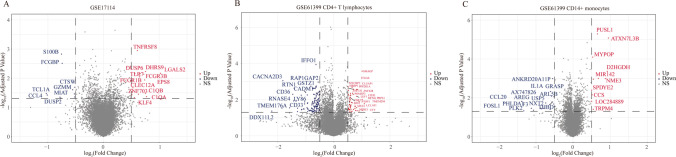
Volcano diagrams of the microarrays. Red points indicate the upregulated genes, while blue points indicate the downregulated genes. Gray points indicate genes without significant difference. **A** GSE17114; **B** GSE61399 CD4 + T lymphocytes; **C** GSE61399 CD14 + monocytes

First, we used the “GCRMA” package to standardize the two-microarray datasets. Supplementary Fig. 6 shows the box plots before and after standardization. Second, we used a PCA diagram to visualize the results of removing the batch effect, as shown in Supplementary Fig. 7. In addition, we used the “limma” package to screen DEGs according to the above criteria, and according to cell grouping, GSE61399 was divided into two groups (CD14 + monocytes and CD4 + T lymphocytes) for difference analysis. Volcano plots of the three groups from the two microarrays are shown in Fig. 1.

### RRA integrated analysis of DEGs

**Fig. 2:**
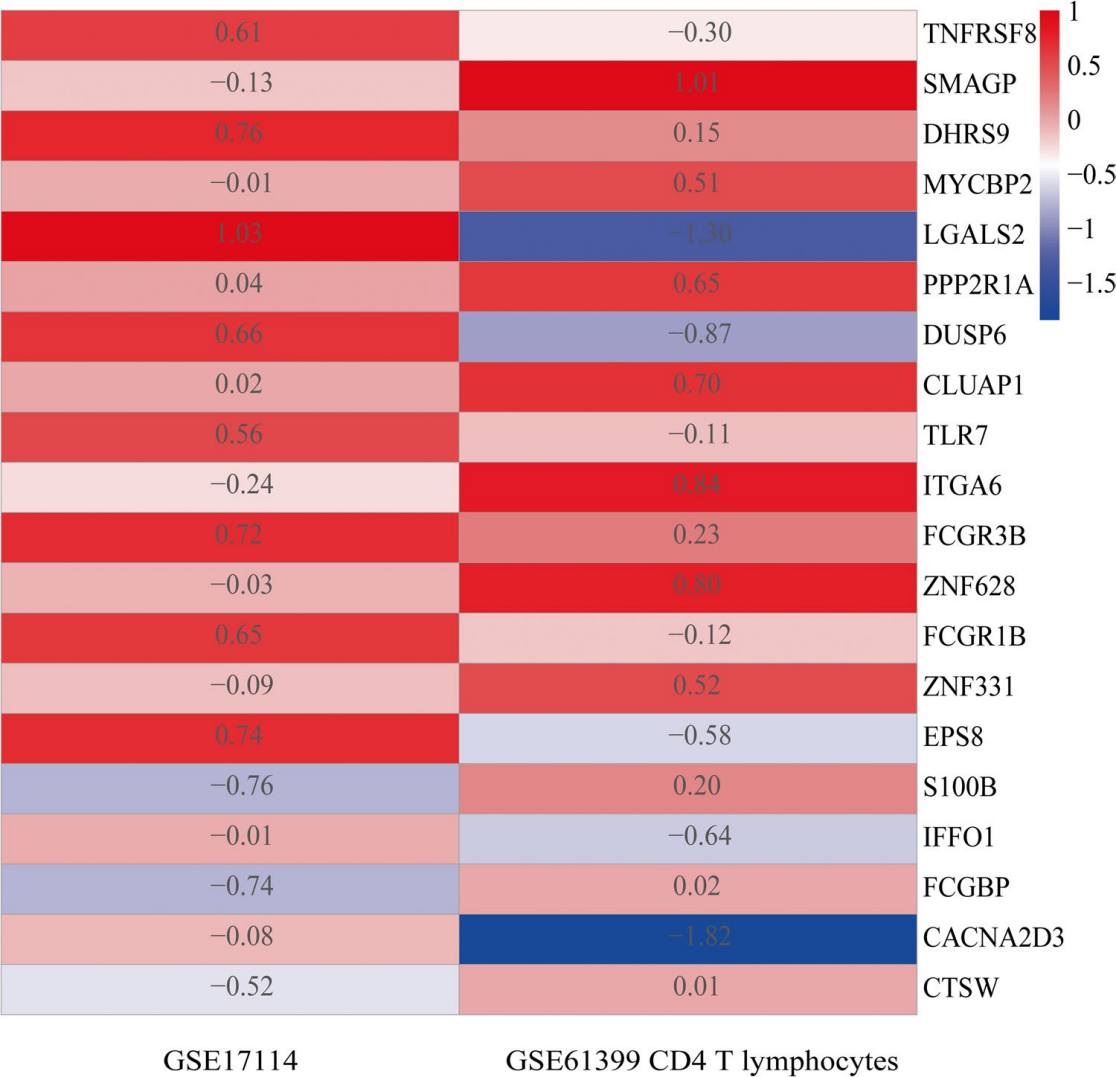
Heatmap of the robust rank aggregation (RRA) analysis. Heatmap of the top 10 upregulated and 10 downregulated genes using the RRA method. Red and blue indicate high and low expression of genes in patients with BD, respectively

We analyzed the integration of GSE17114 and GSE61399 CD14 + monocytes and the integration of GSE17114 and GSE61399 CD4 + T lymphocytes, according to our data and rules set for RRA analysis. After the integrated analysis, no significant differences in the gene expressions of the GSE17114 and GSE61399 CD14 + monocytes were observed. However, 44 significant DEGs (16 upregulated and 28 downregulated) were identified (Supplementary Table 1) between GSE17114 and GSE61399 CD4 + T lymphocytes. The heatmap of the top 10 upregulated and 10 downregulated genes between GSE17114 and GSE61399 CD4 + T lymphocytes is shown in Fig. 2.

### Functional annotation

**Fig. 3:**
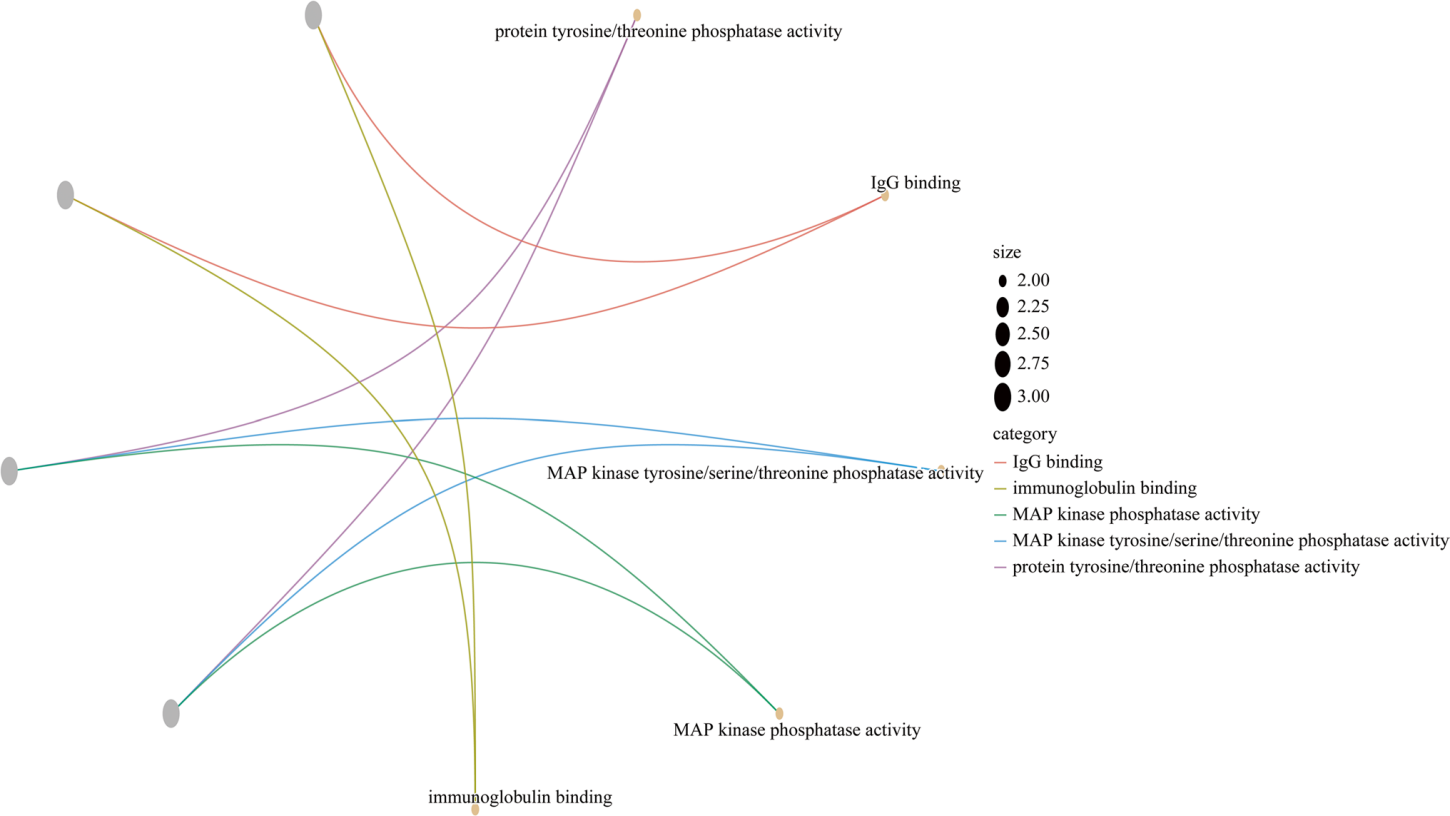
Gene ontology (GO) functional enrichment analysis (cneplot) of differentially expressed genes

We used the 44 DEGs between GSE17114 and GSE61399 CD4 + T lymphocytes to perform GO (molecular function) analysis. The results revealed that protein tyrosine/threonine phosphatase activity (GO:0,008,330; adjusted *P*-value = 0.013) and immunoglobulin binding (GO:0,019,865; adjusted *P*-value = 0.025) were significantly enriched for molecular function. We used a GO cneplot (Fig. 3) to visualize the GO terms.

### Results of protein–protein interaction (PPI) network analysis

**Fig. 4:**
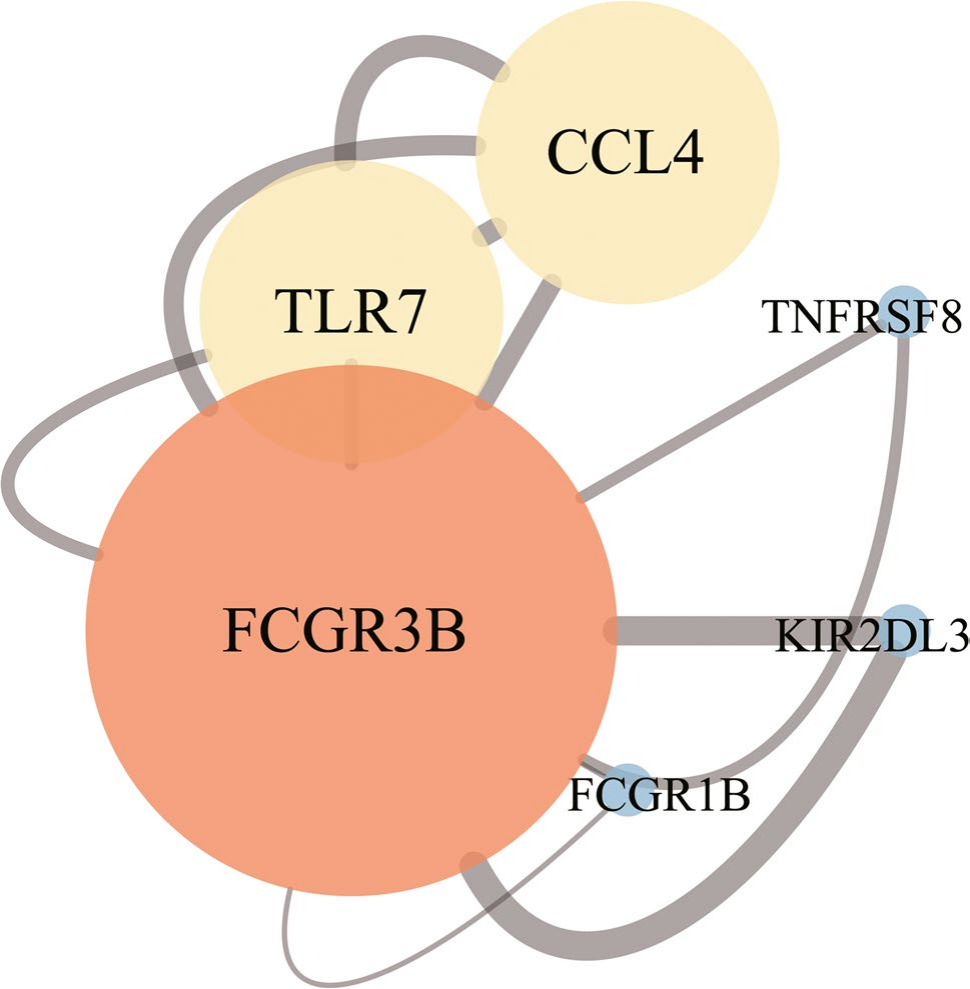
The outcomes of protein–protein interaction (PPI) network analysis

We performed the PPI network analysis using the STRING online database and the significant genes between GSE17114 and GSE61399 CD4 + T lymphocytes as input (Fig. 4). Cytoscape was used to visualize the results. In the PPI network, the genes located in the central node were recognized as key genes that may play crucial regulatory roles in BD. The results showed that the top six genes with the most connections which were *FCGR3B*, *TLR7*, *CCL4*, *FCGR1B*, *TNFRSF8*, *KIR2DL3*, and *FCGR3B* had the largest weight. Therefore, according to RAA and PPI analyses, *FCGR3B* was considered a hub gene.

### The validation of FCGR3B gene

**Fig. 5:**
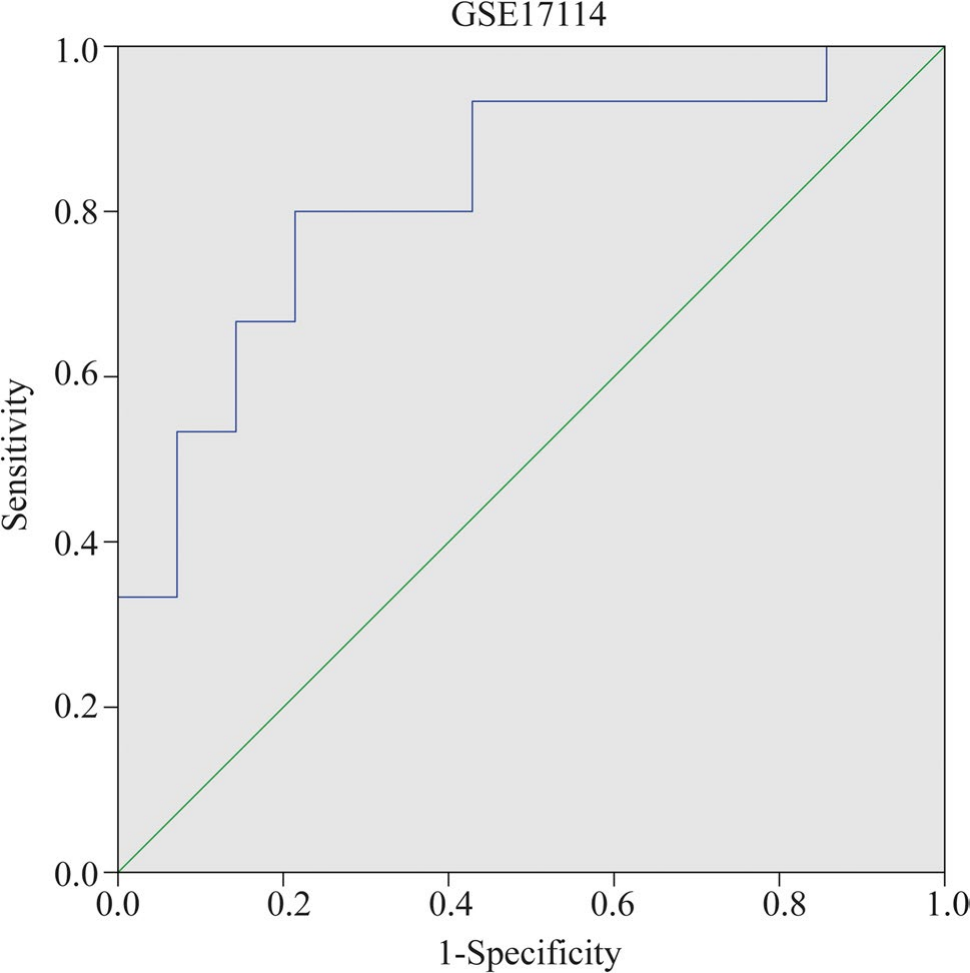
Receiver operating characteristics of *FCGR3B* in GSE17114

To validate the diagnostic value of *FCGR3B* in BD patients, we performed ROC analyses to investigate the sensitivity and specificity of *FCGR3B* for BD diagnosis. The ROC outcomes verified that *FCGR3B* could differentiate between BD patients and healthy controls in GSE17114 (*P* < 0.05), with an AUC of 0.824 (Fig. 5). However, the diagnostic value of *FCGR3B* in GSE61399 and GSE61399 CD4 + T lymphocytes was uncertain (Supplementary Fig. 8A and 8B). This is due to the large difference in the sample size of BD CD4 + T lymphocytes in patients and healthy controls in GSE61399, causing some bias. Our results indicated that expression of *FCGR3B* was related to disease diagnosis and *FCGR3B* could be used as a biomarker in the diagnosis of BD.

## Discussion

BD is a common autoimmune disease and is diagnosed based on recurrent oral ulcers (recurrent at least 3 times within 1 year) [37]. Currently, there is no specific antibody for the diagnosis of BD. The diagnosis mainly depends on medical experience and the invasive skin acupuncture reaction [37]. Thus far, the pathogenesis of BD has not been clarified. Relevant studies suggest that its pathogenesis may be the result of multiple effects of autoimmunity, external environmental factors, and genetic susceptibility [3]. Other studies have reported that BD patients show specific microbiota characteristics [38]. Therefore, there is an urgent need to better understand the pathogenesis of BD to formulate new strategies for the diagnosis and treatment of BD.

In the current study, based on the gene expression profiles obtained from GSE17114 and GSE61399 datasets, *FCGR3B* was identified as the key DEG between the CD4 + T lymphocytes of patients with BD and healthy controls using bioinformatic tools. We explored the biological processes of these DEGs using GO enrichment analysis. The results showed that DEGs were significantly correlated with protein tyrosine/threonine phosphatase activity and immunoglobulin binding. We performed PPI network analysis to identify core genes. Next, we performed ROC analysis to study the sensitivity and specificity of core gene diagnosis of BD, and the results showed that the expression of *FCGR3B* was related to the diagnosis of BD CD4 + T lymphocytes.

The Fc receptor family for immunoglobulin (Ig)G (FCGRs) is mainly expressed on immune effector cells and modulates the response of IgG antibodies. Furthermore, FCGRs mainly mediate immune responses [39–41]. When the regulatory system involved in FCGR becomes dysfunctional, it can lead to the onset or deterioration of autoimmune diseases [39, 40]. FCGRs include three high-affinity FCGRs (FCGRIa, FCGRIb, and FCGRIc) and five low-affinity FCGRs (FCGRIIa, FCGRIIb, FCGRIIc, FCGRIIIa, and FCGRIIIb). The *FCGR3B* gene encodes FCGRIIIb (also known as CD16b), specifically expressed on neutrophils [42]. Previous studies demonstrated that FCGR3 gene copy number variations (CNVs) and single nucleotide polymorphisms (SNPs) are associated with several diseases, especially autoimmune disorders, such as systemic lupus erythematosus [43–45], rheumatoid arthritis [45], ANCA-associated systemic vasculitis (AASV) [43, 46, 47], sarcoidosis [48, 49], and others [50]. Few hypotheses suggest that FCGRIIIb is primarily expressed on neutrophils, and hence its deficiency or variation may obstruct the clearance of immune complexes by neutrophils and enhance the pro-inflammatory effect [47]. Relevant studies have shown that FCGR gene polymorphisms are related to BD, suggesting that FCGR genes may play a role in the pathogenesis of BD [51, 52]. Huang et al. studied the expression of FcγRIIb, FcγRI, and FcγRIII on monocytes, T cells, and other cells in patients with BD and showed that FcγR is abnormally expressed in BD monocytes and is associated with disease progression and might promote the over-activation of monocytes in BD patients [53]. However, Black et al. found that there was no correlation between high or low copy number of *FCGR3B* and BD or its clinical features in the Iranian population [54]. Therefore, the exact role of *FCGR3B* in the pathogenesis of BD remains unclear. The aim of this study was to analyze the microarray data of GSE17114 and GSE61399 using bioinformatics, primarily using RRA, GO enrichment, and PPI network analyses. Our experiments showed that *FCGR3B* may be involved in the pathogenesis of BD CD4 + T lymphocytes. A relevant study has shown that Th1 and Th2 cytokines (IFN-γ and IL-4) differentially regulate the expression of FcγR isoforms with opposite functions, altering the balance of activating and inhibitory signals delivered by FcγRs present on phagocytes [55]. At the same time, previous studies have shown that T lymphocytes are the main infiltrating cell type of the local inflammatory foci in BD [56]. Another study showed that Th1 and Th17 cells cause inflammation through abnormal and persistent cytokine production (IFN-γ, TNF-α, and IL-17) and cytotoxicity mediated by perforin and Fas ligands, leading to gastrointestinal mucosal damage in BD patients [10]. The counts of CD4 + and CD8 + T cells producing cytokines are increased in the circulating blood and inflammatory tissues of BD [7–9]. The verification test indicated that the expression of *FCGR3B* was related to BD diagnosis.

This study has some limitations. First, we did not conduct in vivo tests to verify the outcomes. Second, we need to further study the definite mechanism of the immune response induced by *FCGR3B*. Finally, we did not explore the association of *FCGR3B* with the serological phenotypes (autoantibody profiles) of patients with BD. Although bioinformatics can reveal the internal mechanism, the results of our study need to be further validated by in vivo and in vitro tests and medical analysis.

In summary, we have comprehensively provided a profound understanding of the molecular changes in BD and identified *FCGR3B* as a hub gene. Moreover, GO enrichment analysis revealed that these DEGs were generally enriched in protein tyrosine/threonine phosphatase activity and immunoglobulin binding. However, the mechanism of action of *FCGR3B* has not been fully elucidated. More experiments are needed to verify the results, and more samples from patients with BD and healthy controls need to be collected for additional functional research.

## Supplementary Information

Below is the link to the electronic supplementary material.

Supplementary Fig. 6 (PNG 450 KB)

High Resolution Image (TIF 19.5 MB)

Supplementary Fig. 7 (PNG 569 KB)

High Resolution Image (TIF 15.2 MB)

Supplementary Fig. 8 (PNG 71.9 KB)

High Resolution Image (TIF 4.18 MB)

ESM 4 (DOCX 17.2 KB)
