# Supplementary figures and images for: Identification of novel genes in Behcet’s disease using integrated bioinformatic analysis
Source: Immunol Res. 2022 Apr 2;70(4):461–8. doi: 10.1007/s12026-022-09270-3 (PMC9273552; doi:10.1007/s12026-022-09270-3)

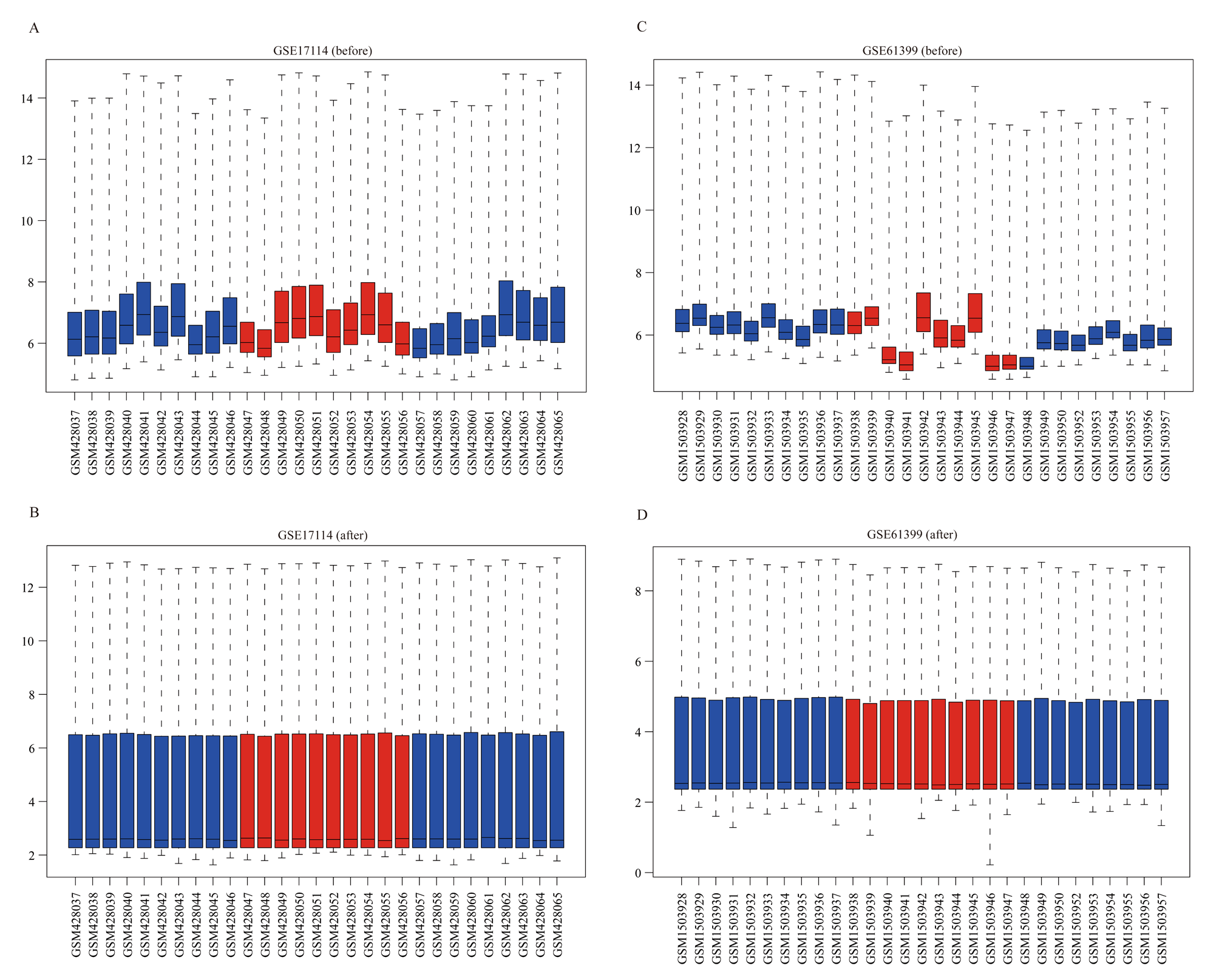

Supplement: Supplementary file 1 — (PNG 450 KB) [file 12026_2022_9270_Fig6_ESM.png]

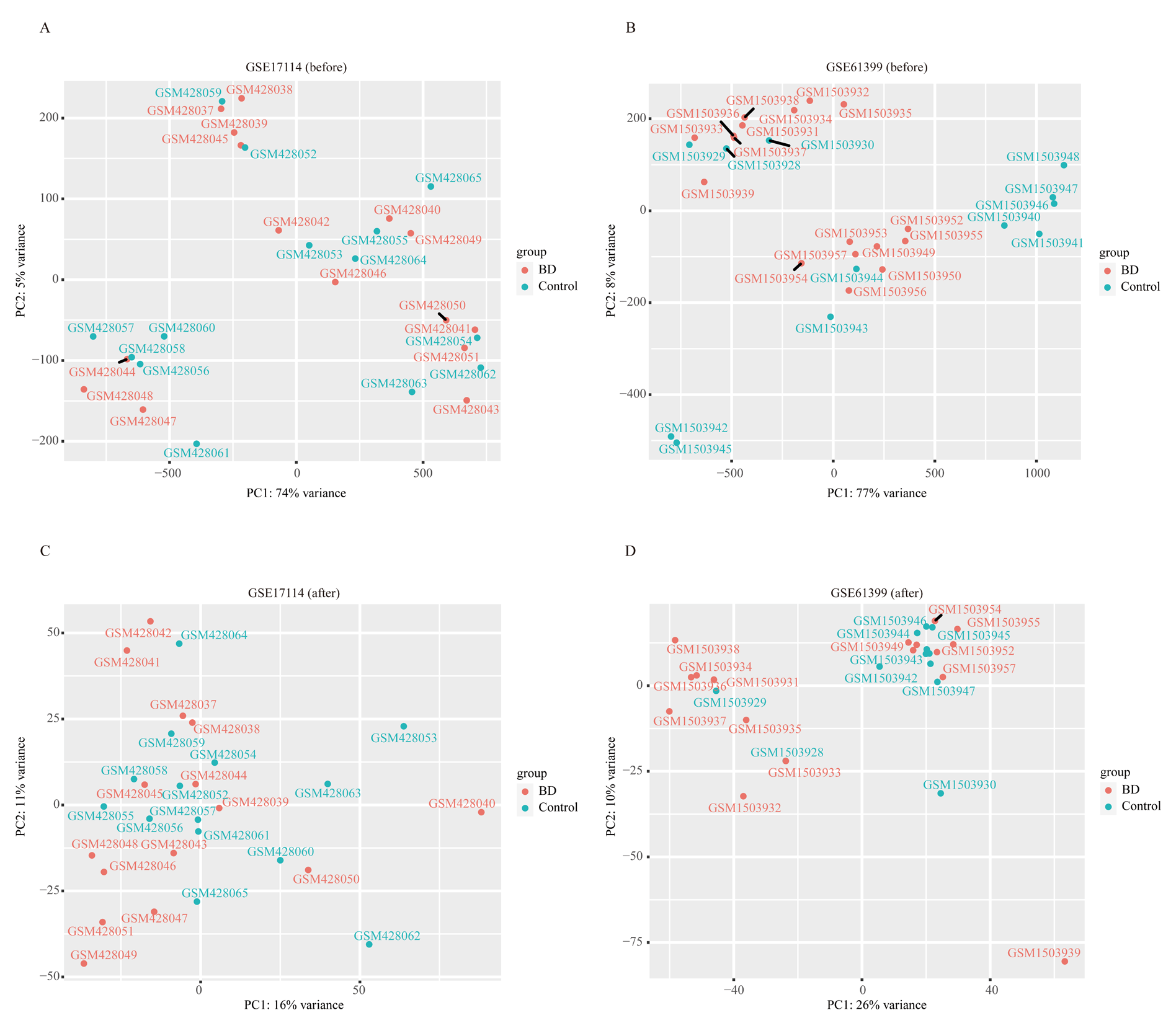

Supplement: Supplementary file 3 — (PNG 569 KB) [file 12026_2022_9270_Fig7_ESM.png]

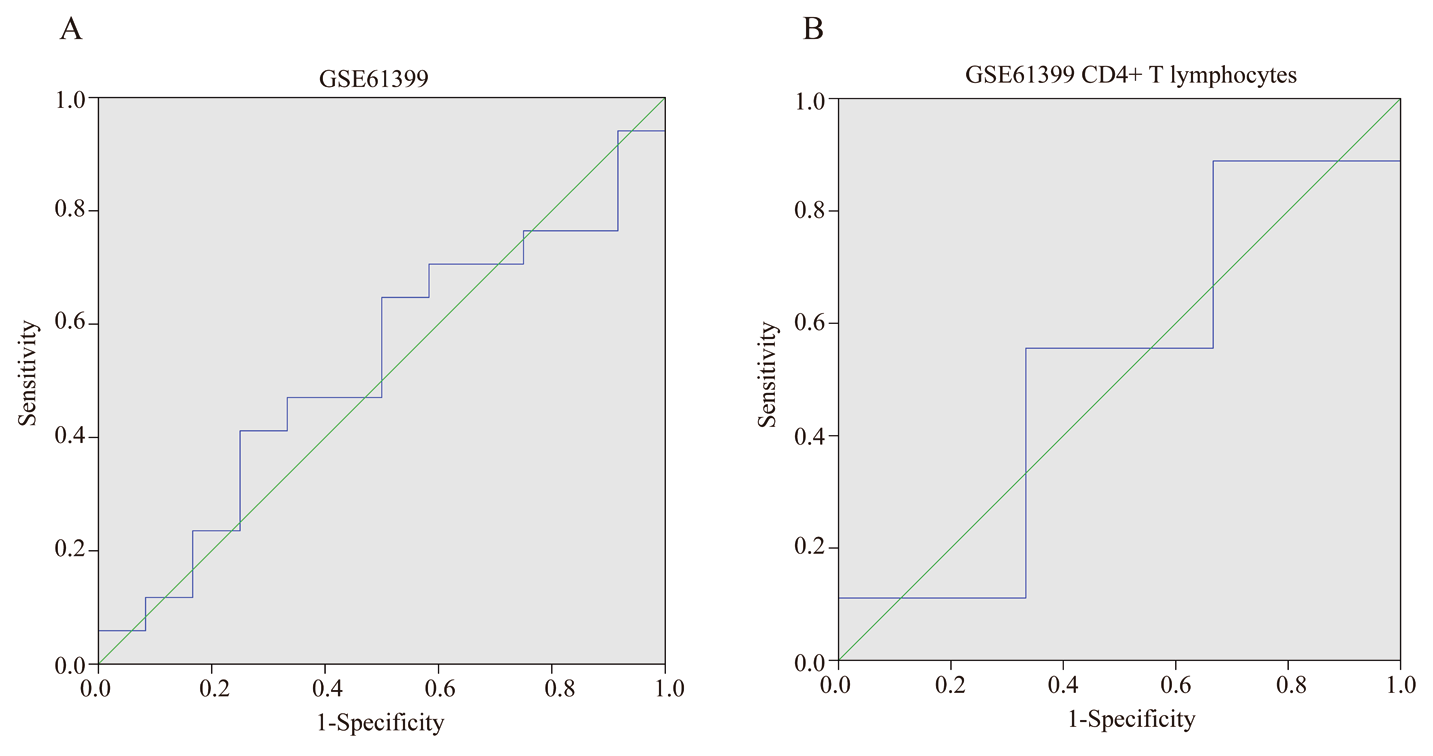

Supplement: Supplementary file 5 — (PNG 71.9 KB) [file 12026_2022_9270_Fig8_ESM.png]
