# Supplementary material for: Identification of novel genes in Behcet’s disease using integrated bioinformatic analysis
Source: Immunol Res. 2022 Apr 2;70(4):461–8. doi: 10.1007/s12026-022-09270-3 (PMC9273552; doi:10.1007/s12026-022-09270-3)
Supplement: Supplementary file 7 — (DOCX 17.2 KB) [file 12026_2022_9270_MOESM4_ESM.docx]

**Supplementary Table 1. The 44 DEGs identified by RRA method between GSE17114 and GSE61399 CD4 + T lymphocytes.**

| **Gene symbol** | **P value** | **Up/down regulated in DM/PM** |
| --- | --- | --- |
| TNFRSF8 | 0.059255959 | up |
| SMAGP | 0.059255959 | up |
| DHRS9 | 0.117620851 | up |
| MYCBP2 | 0.117620851 | up |
| LGALS2 | 0.175094676 | up |
| PPP2R1A | 0.175094676 | up |
| DUSP6 | 0.231677434 | up |
| CLUAP1 | 0.231677434 | up |
| TLR7 | 0.287369125 | up |
| ITGA6 | 0.287369125 | up |
| FCGR3B | 0.342169748 | up |
| ZNF628 | 0.342169748 | up |
| FCGR1B | 0.396079305 | up |
| ZNF331 | 0.396079305 | up |
| EPS8 | 0.449097795 | up |
| TSR2 | 0.449097795 | up |
| S100B | 0.028880487 | down |
| IFFO1 | 0.028880487 | down |
| FCGBP | 0.057550935 | down |
| CACNA2D3 | 0.057550935 | down |
| CTSW | 0.086011342 | down |
| RAP1GAP2 | 0.086011342 | down |
| GZMM | 0.11426171 | down |
| DPY19L3 | 0.11426171 | down |
| TCL1A | 0.142302037 | down |
| LOC100505715 | 0.142302037 | down |
| MIAT | 0.170132325 | down |
| MEF2C | 0.170132325 | down |
| CCL4 | 0.197752573 | down |
| CHKB | 0.197752573 | down |
| RP11-124L9.5 | 0.225162781 | down |
| ZNF808 | 0.225162781 | down |
| DUSP2 | 0.252362949 | down |
| RTN1 | 0.252362949 | down |
| KIR2DL3 | 0.279353077 | down |
| SPTSSA | 0.279353077 | down |
| SUMF1 | 0.306133165 | down |
| LOC100134822 | 0.332703214 | down |
| HEATR3 | 0.359063222 | down |
| MICAL1 | 0.385213191 | down |
| GSTZ1 | 0.411153119 | down |
| RP1-93H18.6 | 0.436883008 | down |
| LINC00893 | 0.462402857 | down |
| B4GALT2 | 0.487712665 | down |
